# Reframing the approach to heterosexual men in the HIV epidemic in sub‐Saharan Africa

**DOI:** 10.1002/jia2.25510

**Published:** 2020-06-26

**Authors:** Tawanda Makusha, Heidi van Rooyen, Morna Cornell

**Affiliations:** ^1^ Human Sciences Research Council Pretoria South Africa; ^2^ MRC/Wits Developmental Pathways for Health Research Unit (DPHRU) School of Clinical Medicine University of the Witwatersrand Johannesburg‐Braamfontein South Africa; ^3^ DSI‐NRF Centre of Excellence in Human Development University of the Witwatersrand Johannesburg‐Braamfontein South Africa; ^4^ Centre for Infectious Disease Epidemiology & Research School of Public Health & Family Medicine University of Cape Town Cape Town South Africa

**Keywords:** Heterosexual men, HIV epidemic, prevention, testing, antiretroviral therapy, sub‐Saharan Africa

Despite the body of evidence on heterosexual men’s inequitable access to HIV prevention, testing and antiretroviral therapy (ART) [1, 2], and poorer viral suppression in sub‐Saharan Africa (SSA), public health responses to address this gap remain surprisingly sparse [3]. Gender stereotypes prevail, implicitly blaming men for infecting women with HIV, and for their own health outcomes due to “poorer health‐seeking behaviour” [4]. These generalizations about men come at a cost, as neither men nor women benefit when men are portrayed largely as vectors of disease, and when the health needs of women and men are seen as competing rather than complementary. Recent evidence suggests that men care about their health and will participate in HIV prevention, testing and treatment programmes when these are appropriately targeted [5]. This viewpoint argues for a reframing of the approach to heterosexual men in the HIV epidemic in SSA.

The gendered nature of health services in SSA has been well described [1, 3, 4, 6, 7]. Given women’s biological and social vulnerability to HIV infection, research, programmes and policies have primarily focused on the needs of women [8]. When programmes have included heterosexual men, whether intentional or not, they have frequently been depicted as the *problem* (i.e. transmitting HIV), and the health outcomes of women and children have been prioritized [9]. Consequently, the health needs of men in SSA and generally across the world have been largely ignored [1, 3, 10]. There are two compelling reasons why the health and HIV risks of heterosexual men should be addressed urgently: like women, men have the right to health; and to reach the ambitious UNAIDS targets of 90:90:90, we need a response that is based on public health and gender inclusiveness rather than gender bias. Given men’s higher AIDS‐related morbidity and mortality in the context of a limited focus on men, HIV‐positive men represent a new vulnerable population in the AIDS epidemics of sub‐Saharan Africa [3]. The exclusion of heterosexual men from targeted HIV prevention, testing and treatment strategies constrains the ability of HIV‐positive men to manage the risks associated with their health and increases the gender gap in HIV survival [1, 4].

A successful HIV response requires a shift from portraying men as the “problem” to acknowledging that, like women, men are vulnerable to HIV infection due to individual, social and structural drivers. We recently undertook a study in a peri‐urban region of KwaZulu‐Natal province of South Africa, an area where high levels of poverty, unemployment and alcohol consumption co‐exist alongside high HIV prevalence rates. A total of 6993 men participated in male‐focused community‐based HIV and non‐communicable diseases screening from August 2017 to June 2019. In contrast to widely cited generalizations about men’s poorer health‐seeking behaviour, we found that men were concerned about their own and their families’ health. Out of 6988 men who consented to HIV screening, 6740 (97%) gave consent for an HIV test [4]. Notably, 80% of the men felt blamed for the HIV epidemic, and unsupported when they did access healthcare services [4]. This study also highlighted the inadequacy of HIV prevention for men which focuses solely on HIV, outside of the broader contexts which shape HIV risk and vulnerability.

Our work confirms that men, like women, are not a homogenous group. The 2017 South African National HIV Prevalence, Incidence, Behaviour and Communication Survey found that age, race, education, employment and locality type were all significant predictors of new HIV infection among men aged 15 years and older [11]. Thus, like women, men are vulnerable to HIV infection due to individual, social and structural drivers, which function in tandem with risky sexual behaviours to increase their risk of HIV infection. Despite these challenges, these men belong to families; they are partners and they are fathers. They can survive and thrive when they live in families and communities that are supportive, caring, loving and resilient.

The news is not all bleak, however. Recently, there is some sense that the narrative has shifted. International agencies such as UNAIDS, the World Health Organization and PEPFAR have an increased focus on men and HIV. Some countries have developed, or are formulating, national strategic plans on men and HIV [12]. Using Demographic and Health Surveys data, researchers recently characterized the “missing men” in six African countries, highlighting the particular need to reach poor single men without children in rural areas [2]. Others are researching the preferences of men, to inform the development of effective programmes. These are important steps towards ensuring that men are part of the HIV response.

The landmark IAS Forum on Men and HIV prior to the 10th IAS Conference in Mexico represented a turning point in challenging the prevailing discourse on men. Building on the momentum created by the Forum, it is time to reframe the approach to heterosexual men in the HIV epidemic. Men and women should not be seen as competing populations. Like women, men have the right to health, HIV care and treatment, and their poorer access to HIV care cannot be reduced to individual behaviour. HIV interventions for both men and women must be guided by evidence. We need to watch our language: no more “men as a problem” or “men as the vectors”. HIV interventions should improve heterosexual men’s health for their own sake, not only to improve outcomes for women and children. Future HIV/AIDS conferences must include heterosexual men as a vulnerable population. In getting the frame right in the way we view men, we have the chance to address the biggest gap in the response to HIV in SSA.

## COMPETING INTERESTS

The authors declare that they have no competing interests.

## AUTHORS’ CONTRIBUTIONS

TM conceptualized and drafted the paper, HvR provided comments and edits, MC provided guidance on the key messages and edited drafts. All authors approved the final version.

## ABBREVIATIONS

AIDS, Acquired immunodeficiency syndrome; ART, Antiretroviral therapy; HIV, Human immunodeficiency virus; SSA, sub‐Saharan Africa.
